# RETRACTION: Potentiality of Forkhead Box Q1 as a Biomarker for Monitoring Tumor Features and Predicting Prognosis in Non‐Small Cell Lung Cancer

**DOI:** 10.1002/jcla.70237

**Published:** 2026-04-13

## Abstract

**RETRACTION**: L. Li, B. Xu, H. Zhang, J. Wu, Q. Song, J. Yu, “Potentiality of Forkhead Box Q1 as a Biomarker for Monitoring Tumor Features and Predicting Prognosis in Non‐Small Cell Lung Cancer,” *Journal of Clinical and Laboratory Analysis* 34, no. 1 (2019): e23031, https://doi.org/10.1002/jcla.23031.

The above article, published online on 12 November 2019 in Wiley Online Library (wileyonlinelibrary.com), has been retracted by agreement between the journal Editor‐in‐Chief, Rong Fu; and Wiley Periodicals, LLC. The retraction has been agreed due to concerns raised by a third party, which identified compromised data and systematic issues in the published article. The authors and their institutes were unresponsive when these concerns were brought to their attention. Accordingly, the article is retracted as the editors have lost trust in the reliability of the data and the results presented.

**RETRACTION**: L. Li, B. Xu, H. Zhang, J. Wu, Q. Song, J. Yu, “Potentiality of Forkhead Box Q1 as a Biomarker for Monitoring Tumor Features and Predicting Prognosis in Non‐Small Cell Lung Cancer,” *Journal of Clinical and Laboratory Analysis* 34, no. 1 (2019): e23031, https://doi.org/10.1002/jcla.23031.

The above article, published online on 12 November 2019 in Wiley Online Library (wileyonlinelibrary.com), has been retracted by agreement between the journal Editor‐in‐Chief, Rong Fu; and Wiley Periodicals, LLC. The retraction has been agreed due to concerns raised by a third party, which identified compromised data and systematic issues in the published article. The authors and their institutes were unresponsive when these concerns were brought to their attention. Accordingly, the article is retracted as the editors have lost trust in the reliability of the data and the results presented.

